# A human glomerular SAGE transcriptome database

**DOI:** 10.1186/1471-2369-10-13

**Published:** 2009-06-05

**Authors:** Jenny Nyström, Wolfgang Fierlbeck, Anna Granqvist, Stephen C Kulak, Barbara J Ballermann

**Affiliations:** 1 Department of Medicine, University of Alberta, Edmonton, Alberta, Canada; 2 Department of Nephrology, Göteborg University, Gothenburg, Sweden; 3 Department of Nephrology, University of Frankfurt/Main, Frankfurt, Germany

## Abstract

**Background:**

To facilitate in the identification of gene products important in regulating renal glomerular structure and function, we have produced an annotated transcriptome database for normal human glomeruli using the SAGE approach.

**Description:**

The database contains 22,907 unique SAGE tag sequences, with a total tag count of 48,905. For each SAGE tag, the ratio of its frequency in glomeruli relative to that in 115 non-glomerular tissues or cells, a measure of transcript enrichment in glomeruli, was calculated. A total of 133 SAGE tags representing well-characterized transcripts were enriched 10-fold or more in glomeruli compared to other tissues. Comparison of data from this study with a previous human glomerular Sau3A-anchored SAGE library reveals that 47 of the highly enriched transcripts are common to both libraries. Among these are the SAGE tags representing many podocyte-predominant transcripts like WT-1, podocin and synaptopodin. Enrichment of podocyte transcript tags SAGE library indicates that other SAGE tags observed at much higher frequencies in this glomerular compared to non-glomerular SAGE libraries are likely to be glomerulus-predominant. A higher level of mRNA expression for 19 transcripts represented by glomerulus-enriched SAGE tags was verified by RT-PCR comparing glomeruli to lung, liver and spleen.

**Conclusion:**

The database can be retrieved from, or interrogated online at http://cgap.nci.nih.gov/SAGE. The annotated database is also provided as an additional file with gene identification for 9,022, and matches to the human genome or transcript homologs in other species for 1,433 tags. It should be a useful tool for in silico mining of glomerular gene expression.

## Background

Renal glomeruli are highly specialized capillary tufts that produce a nearly protein-free ultrafiltrate of plasma at a rate of about 30 plasma volumes daily. Several hereditary, immune-mediated and metabolic disorders cause glomerular injury, proteinuria, and can lead to renal failure. The three intrinsic glomerular cell types, podocytes, mesangial cells, and glomerular endothelial cells (EC) are highly specialized. Podocytes extend an elaborate array of actin-rich foot processes around the exterior of the glomerular capillary loops, forming a scaffold with nephrin-based filtration slit diaphragms spanning the space between adjacent foot processes [1]. Mesangial cells are pericyte-like cells that, unlike most other pericytes, form an interstitium *within *the intracapillary space [2]. Glomerular EC are packed with transcellular fenestrae ringed by actin [3,4]. The fenestrae serve the high glomerular capillary wall hydraulic conductivity [5], while a glycocalyx covering the glomerular EC and podocytes together with the podocyte filtration slit diaphragm impede the movement of plasma proteins [6-8] across the glomerular capillary wall.

Transcriptome and proteomic approaches are helping to define genes highly expressed and/or enriched in glomeruli [9-12]. For instance, Sau3A-anchored SAGE databases have been built with RNA extracted from microdissected nephron segments, and enrichment of several glomerular transcripts relative to other nephron segments has been reported [9]. Furthermore, many proteins uniquely expressed by, or enriched in podocytes have been identified over the past decade and their specific functions are increasingly well defined [13]. Finally, by analysis of ESTs enriched in glomeruli, ehd3 was shown to be the first transcript expressed exclusively by glomerular EC [12].

The current study sought to extend previous transcriptome-based work by building a human glomerular LongSAGE database that can be interrogated directly online. SAGE is based on the principal that a small (here 17 bp) tag sequence immediately 3' of an "anchoring" restriction site is a unique identifier of each transcript [14]. The frequency of specific SAGE tags relative to the total pool of tags reflects their abundance in the source mRNA. In silico comparison of SAGE libraries from diverse tissues can then be used to discover differential expression of transcripts [15]. The level of expression of any specific transcript can also be probed in silico by interrogating SAGE libraries with the transcript's unique SAGE tag sequence.

We report here the gene expression profile of transcripts for human glomeruli and compare them to pooled SAGE libraries for non-glomerular tissues and cells. Many of the most highly enriched glomerular transcripts reported here were previously found in a Sau3A-anchored glomerular library [9]. Nonetheless, the current SAGE database contains additional glomerulus-enriched transcript tags, and since it is NlaIII-anchored it now allows direct comparison with many non-renal SAGE libraries. The data should serve as a useful resource for investigators studying glomerular gene expression.

## Construction and content

### Cells and Tissues

**Figure 1:**
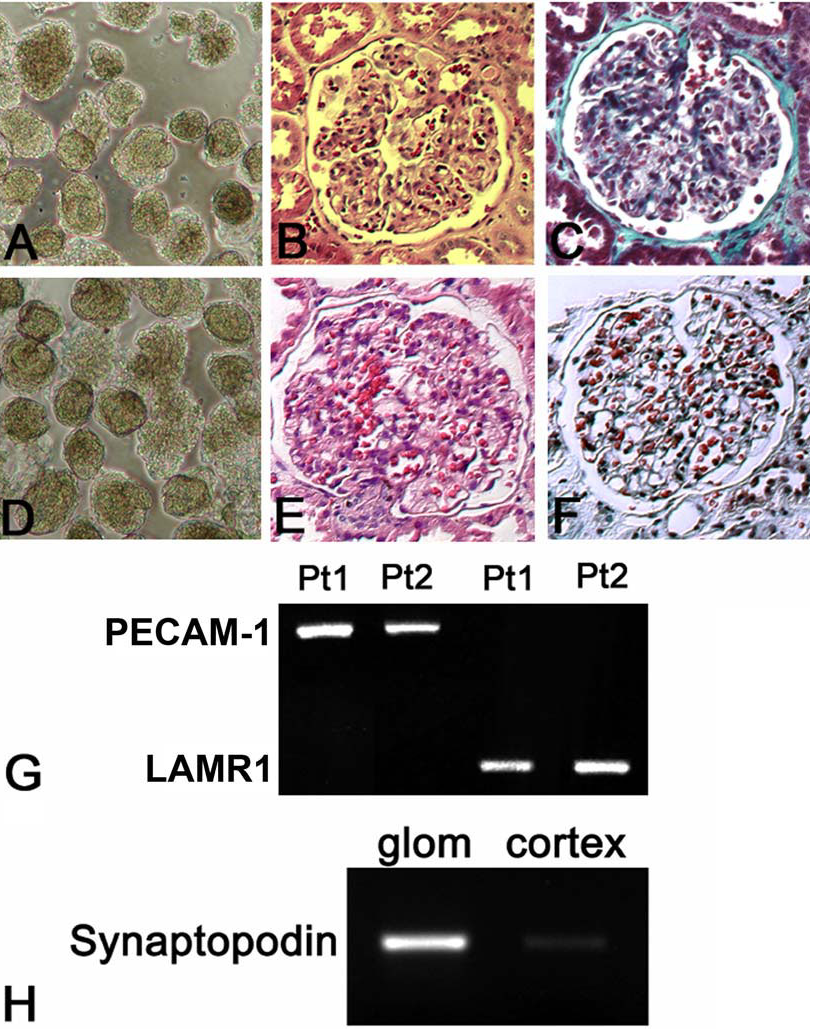
**Human Kidney Source Material**. Isolated glomeruli **(A, D)**, Hematoxylin & Eosin Stain **(B, E) **and Masson Trichrome Stain **(C, F) **for patient 1 **(A, B, C) **and patient 2 **(D, E, F)**. RT-PCR for PECAM-1 and the 67 kDa non-integrin laminin receptor LAMR1 for patient 1 (pt 1) and patient 2 (pt2) **(G)**. Enrichment of the synaptopodin mRNA abundance, determined by RT-PCR, in glomeruli relative to whole kidney cortex from patient 2 **(H)**.

Human kidney tissue was obtained from the uninvolved portion of tumor nephrectomy specimens (Human Subjects Protocols: #6196 University of Alberta and #155/97 University of Frankfurt). Patient 1 was a 45 year old Caucasian female, patient 2 a 72 yo Caucasian male. Renal tissue was collected only from patients in whom the serum creatinine was within normal limits, and in whom diabetes mellitus, hypertension, and proteinuria were absent. Specific parameters were not collected for individual patients. The relative purity of isolated glomeruli and the normal histological appearance of kidney cortex used in this study are shown in Figure 1. That the cDNA template used for SAGE contained mRNA representing glomerular capillary endothelium is shown by RT-PCR amplification of PECAM-1 (836 bp) and the non-integrin laminin receptor LAMR1 (256 bp). Greater synaptopodin transcript abundance in glomerular, compared to whole kidney cortex mRNA from the same specimen also shows appropriate enrichment of the source mRNA in glomerular podocyte transcripts (Figure 1). Amplification of the long PECAM-1 sequence furthermore shows that the source mRNA was intact. Sufficient mRNA for construction of the SAGE library was only obtained from patient 2. The integrity of this source material was also verified by Agilent 2100 microfluidics analysis (data not shown).

### Human Glomerular SAGE Library Construction

For SAGE, human glomeruli were isolated by sieving in ice-cold phosphate buffered saline (PBS) from the kidney of a 72-year old male using minor modifications of the protocol for rat glomeruli [16,17]. Glomeruli were immediately placed into RNA-Protect (Qiagen, Valencia, CA) followed by isolation of 4.7 μg total RNA with the RNeasy kit (Qiagen). A SAGE library was then custom-constructed by Genzyme Corporation (Framingham, MA) using the "long" SAGE protocol, producing 17 bp SAGE tags with the CATG (NlaIII) anchoring restriction site [18]. A total of 2304 clones containing concatenated ditags were sequenced, resulting in 48,926 tags. Of these, 1,361 were derived from duplicate ditags. Tags from duplicate ditags were not removed [19]. Twenty-one tags were removed as they contained ambiguous (N) nucleotides leaving 48,905 tags and 22,907 unique long SAGE tags for analysis.

Tag sequences and their absolute counts in 115 distinct "long" SAGE libraries were retrieved from cgap.nci.nih.gov/SAGE (98,944,923 tags). All "long" SAGE Libraries available before July 1, 2008 were included without selection. Tissues and cells represented include normal brain, breast, skin, pancreas, bladder, gallbladder, uterus, vein, testis, white blood cells, lung macrophages, embryonic stem cells as well as malignant tumors including colon and lung adenocarcinoma, melanoma, among others. The frequency of each tag (count/total tag number) was calculated for each of the 115 libraries and expressed as tags per million (TPM). The mean TPM for the non-glomerular libraries (Pool TPM) is reported. The frequency ratio of the glomerular: Pool TPM was then calculated to establish degree of enrichment of specific tags in glomeruli (Ratio G: P). Statistical comparison of the pooled libraries with the current glomerular SAGE library was based on Chi-square analysis using absolute tag counts [20]. Comparison to a human kidney SAGE library (SAGE_Kidney_normal_B_1, from cgap.nci.nih.gov/SAGE) was based on the short (10 bp) tag sequences.

For each SAGE tag, identification was based on the "Hs_long.best_gene.gz" database found at ftp://ftp1.nci.nih.gov/pub/SAGE/HUMAN/. The SAGE Genie algorithm for identifying the best gene match for SAGE tags was reported by Boon et al. [21]. For some tags, the Blast *n *algorithm at http://blast.ncbi.nlm.nih.gov/Blast.cgi, was used to match tag sequences that could not be assigned by the "Hs_long.best_gene.gz" database. For these, the SAGE tag had to be in the +/+ orientation with the corresponding mRNA or EST, and fully match the 17 bp sequence immediately 3' of the NlaIII site nearest the Poly(A)^+ ^tail or a stretch of > 8 A's as previously reported [22]. Positive identification based on this latter search strategy is indicated in additional file 1 by asterisks.

### RT-PCR Analysis

For quantitative RT-PCR, glomeruli were microdissected from distinct pre-transplant kidney biopsy specimens obtained from three separate donors aged 57, 59 and 63 at the University of Göteborg (Human Subjects Protocol #653-05). Immediately after biopsy, one half of one biopsy core was placed into 0.5 ml of ice-cold PBS containing 100 U RNAse inhibitor (RNAsin) (Applied Biosystems, Foster City, CA, USA). Four to fifteen glomeruli were isolated using a stereomicroscope (Zeiss, Jena, Germany) followed by extraction of total RNA. cDNA was generated from glomerular RNA with SuperScript™ III RT (Invitrogen, Carlsbad, CA, USA). Human kidney, spleen, lung and liver mRNA was purchased from Invitrogen/Ambion (Carlsbad, CA). Reactions without RT for each primer set served as controls. PCR cycling was performed with 100 ng template (94°C – 3 min; 35 cycles: 94°C – 30 sec; 55°C – 30 sec; 68°C – 30 sec plus 1 min for each kilobase pair (kbp) of PCR product to be amplified; 72°C – 7 min). Quantification of gene expression was performed according to the delta Ct method (DeltaCt2/DeltaCt1), as described by others [23], and by this laboratory [22].

### Human Glomerular SAGE Database Content

The complete human glomerular SAGE library was deposited in the Gene Expression Omnibus http://www.ncbi.nlm.nih.gov/geo/ repository (record GSE8114, Accession # GSM199994) and in the SAGE Genie collection http://cgap.nci.nih.gov/SAGE as "LSAGE_Kidney_Glomeruli_Normal_B_bjballer1". It consists of 22,907, unique 17 bp tag sequences and the absolute tag count for each sequence. The total tag count in the library is 48,905. The library is also appended in spreadsheet format with tag identification (additional file 1).

## Utility

### Retrieval of Highly Enriched Glomerular Transcripts

The transcripts most highly enriched in human glomeruli identified by this study are shown in Tables 1 and 2 and Additional files 3 and 4. Of the 22,907 tags, 291 were observed with an absolute count of 4 (81 TPM) or greater and enriched more than 10-fold relative to pooled non-kidney SAGE libraries. For 84 of these no reliable match to a known cDNA sequence was found, and a match to incompletely defined ESTs was observed for 8 others. The tags representing Aldolase B, uromodulin, glutamyl aminopeptidase, glutathione peroxidase, and SLC25A45 were excluded from this set because they were not enriched relative to whole kidney. They likely represent transcripts expressed at very high levels in contaminating tubules. Several highly expressed transcripts produced more than one unique tag, which is common and usually reflects priming from internal poly A^(+) ^runs or alternatively spliced transcripts. After removal of such redundant tags, 133 well-characterized tags highly enriched in glomeruli were established (Tables 1 and 2 and Additional files 3 &4).

**Table 1:** Transcripts represented by SAGE tags enriched 30 fold or more in glomeruli

Symbol	Gene Name	NlaIII long Tag	Sau3A Tag	G:TPM Sau3A	G:TPM NlaIII	P:TPM NlaIII	K:TPM NlaIII	Ratio G: P	Ratio G: K	Link SAGE Genie	Link Ref
NPHS2	Nephrosis 2, idiopathic, steroid-resistant (podocin)	TTTCCGTGACTCATCTA	CCTCACTGAA	1,534	2,287	0	48	Infinity	48	NPHS2	Ref:NPHS2
SOST	Sclerosteosis	ACATATGAAAGCCTGCA	TCGAGGAGAC	158	82	0	0	Infinity		SOST	
MME	Membrane metallo-endopeptidase	CTGCAGTGTTCGAGTGG	TATAAAGCGA	271	102	0	0	4446		MME	Ref:MME
CLIC5	Chloride intracellular channel 5	AATCTGAACCAATTACC	CAGTCATCTG	1,038	3,593	5	24	719	150	CLIC5	Ref:CLIC5
NPHS1	Nephrosis 1, congenital, Finnish type (nephrin)	TAAACATAAGTATGCTC			1,041	2	0	549		NPHS1	Ref:NPHS1
TCF21	Transcription factor 21	CGAGTGCTGAGCAAGGC	ATAGGATAGC	451	817	2	0	514		TCF21	Ref:TCF21
CDKN1C	Cyclin-dependent kinase inhibitor 1C (p57, Kip2)	CCGCTGCGGGGCCCTGG	AGCGCCTGAG	383	490	1	0	506		CDKN1C	Ref:CDKN1C
DDN	Dendrin	TGAACTTGGCCACATCA			306	1	0	373		DDN	Ref:DDN
THYMU2010816	CDNA FLJ36413 fis, clone THYMU2010816	TATCACTGGGGAGGGAA	ATTAGTCAAT	203	449	2	0	285		THYMU2010816	
FLJ22271	CDNA: FLJ22271 fis, clone HRC03191	GCTTTGTCGCAACGCTC			82	0	0	263		FLJ22271	
PTPRO	Protein tyrosine phosphatase, receptor type, O	GATATACAACAGAAAAC	AACTGTGTAA	226	755	4	0	215		PTPRO	Ref:PTPRO
FGF1	Fibroblast growth factor 1 (acidic)	TAAAGGCCTTTAATAAG	TTCTTAGAAG	474	102	0	24	210	4	FGF1	Ref:FGF1
PTHR1	Parathyroid hormone receptor 1	TGACCAGGCGCTGGGGG			1,143	8	96	141	12	PTHR1	Ref:PTHR1
CLDN5	Claudin 5	GTAGGCGGCTGCCTCTT			82	1	0	127		CLDN5	
LRRC2	Leucine rich repeat containing 2	GGACTGCACTCCAGCCT			82	1	0	108		LRRC2	
AL049990	MRNA; cDNA DKFZp564G112 (from clone DKFZp564G112)	ACTTGGAAATAAACAAA			143	1	0	105		AL049990	
HTRA1	HtrA serine peptidase 1	ACCGACAGGCCAAAGGA	CGCAGGCAGA	1,398	143	1	0	105		HTRA1	
FAM65A	Family with sequence similarity 65, member A	GCTGCTGTCAGCACCCA	AGGCTGTTGT	316	204	2	0	99		FAM65A	
SEMA3G	Semaphorin 3G	ACTGCCCCTGAGCTCTG			674	7	24	94	28	SEMA3G	
SPOCK1	Sparc/osteonectin, cwcv and kazal-like domains proteoglycan 1	AGAATACCTTAATACTG	TTTAATACTT	226	122	1	0	92		SPOCK1	
CRB2	Crumbs homolog 2 (Drosophila)	TGCAGCAGTGGCAGCCT			367	4	0	89		CRB2	
ENPEP	Glutamyl aminopeptidase (aminopeptidase A)	GCCTGGAATTGGATACA	ATATGAATTA	632	143	2	96	79	1	ENPEP	Ref:ENPEP
SLC45A1	Solute carrier family 45, member 1	GGCGTGGACATCTCTCT			143	2	0	77		SLC45A1	
TNNT2	Troponin T type 2 (cardiac)	ATGCATTTTGGGGGTTA	TGCTCCTCGC	338	184	2	24	76	8	TNNT2	
CD34	CD34 molecule	GGACCAGGTCTTGGAGC			102	1	24	75	4	CD34	Ref;CD34
EFNB1	Ephrin-B1	AGGGAAGAGGAAAGTGC			102	2	0	68		EFNB1	Ref:EFNB1
IL1RL1	Interleukin 1 receptor-like 1 (ST2 Protein)	AGGGCAGGGACATCATC	TTTGTAGACT	158	82	1	0	64		IL1RL1	
ST6GALNAC3	ST6 (alpha-N-acetyl-neuraminyl-2,3-beta-galactosyl-1,3)-N-	TGATGCCCTTGAACACC			286	5	0	63		ST6GALNAC3	Ref:ST6GALNAC3
	acetylgalactosaminide alpha-2,6-sialyltransferase 3										
NTNG1	Netrin G1	TGTAACAGCCCCCTCTA			102	2	0	63		NTNG1	
SPOCK2	Sparc/osteonectin, cwcv and kazal-like domains proteoglycan 2	CATAAAGGAAATCAAAT	TGGTAGGTTG	248	1,123	18	0	61		SPOCK2	
ABLIM2	Actin binding LIM protein family, member 2	ACACGCCAAGTCCCGTT			122	2	0	60		ABLIM2	Ref:ABLIM2
MGC16291	Hypothetical protein MGC16291	TGGGTCAAACTCTGAAA			122	2	24	58	5	MGC16291	
BCAM	Basal cell adhesion molecule (Lutheran blood group)	CCCGCCCCCGCCTTCCC	ACGTGGTATC	632	3,205	55	788	58	4	BCAM	Ref:BCAM
TPPP3	Tubulin polymerization-promoting protein family member 3	GTGACCCCAAGGCCAGT			82	1	0	57		TPPP3	
C4orf31	Chromosome 4 open reading frame 31	TACATAAAATTAAAGAG	TAATCTAAGT	226	429	8	0	57		C4orf31	
GJA5	Gap junction protein, alpha 5, 40kDa	GACCATTCCTCGGAGTA	AATCTTTGAT	203	122	2	24	55	5	GJA5	RefGJA5
ITGB8	Integrin, beta 8	TCTTGTATCAATGGCAG			633	12	0	52		ITGB8	Ref:ITGB8
TSPAN2	Tetraspanin 2	CCAAGGCACTGAATTAA			143	3	0	49		TSPAN2	
WT1	Wilms tumor 1	CTGGTATATGGCTTCAA	TTACAAGATA	406	143	3	0	49		WT1	Ref:WT1
COL4A3	Collagen, type IV, alpha 3 (Goodpasture antigen)	TGCATTATTTTCCAGAT			122	3	0	48		COL4A3	REF:COL4A3
ALS2CL	ALS2 C-terminal like	CGATGCTGACGGGACCC			796	17	0	47		ALS2CL	
PLCE1	Phospholipase C, epsilon 1	AACGAACGTGGCTGTAT			163	4	0	46		PLCE1	Ref:PLCE1
WFS1	Wolfram syndrome 1 (wolframin)	ACCCTCCTGTCCAGCAG			204	4	0	46		WFS1	
PCOLCE2	Procollagen C-endopeptidase enhancer 2	ATGGAGGTATGAGGCCT	TATGTTCTCT	271	408	9	24	45	17	PCOLCE2	
MRGPRF	MAS-related GPR, member F	AGGACCCACTGGGCAGC	CTCTTAAGGC	316	327	8	0	43		MRGPRF	
SPTB	Spectrin, beta, erythrocytic	CAATCTGGGGCTGGCCC			82	2	0	42		SPTB	Ref:SPTB
ATP10A	ATPase, class V, type 10A	TCCTCTGCGCCAGGGGA			204	5	0	41		ATP10A	
USHBP1	Usher syndrome 1C binding protein 1	TCATAAACTGTCCTGGA			122	3	0	40		USHBP1	
MAP6	Microtubule-associated protein 6	TACAGTAGTCTTGCTGG			327	8	0	39		MAP6	
NFASC	Neurofascin homolog (chicken)	AGCAATGAAAAGGCCAA			184	5	0	39		NFASC	
PLA2R1	Phospholipase A2 receptor 1, 180kDa	AATTTTGCAAAAAGGAA			82	2	0	39		PLA2R1	
C1QL1	Complement component 1, q subcomponent-like 1	CGCGGCGGCGACGGCAC			184	5	24	39	8	C1QL1	
TMEM178	Transmembrane protein 178	CTTGTTAAATTTTAATG			102	3	24	39	4	TMEM178	
TP53I11	Tumor protein p53 inducible protein 11	TACCCCAAGGCCTGATG			102	3	0	37		TP53I11	
SYNPO	Synaptopodin	ATATTAGGAAGTCGGGG	CATTTCTACC	519	592	16	0	37		SYNPO	Ref:SYNPO
DACH1	Dachshund homolog 1 (Drosophila)	TAGGACCTATGAAAATT			82	2	0	37		DACH1	
TTMA	Two transmembrane domain family member A	CTTTATTGAGTGTTATC			225	6	0	37		TTMA	
FABP1	Fatty acid binding protein 1, liver	ACATTGGGTGACATTGT			102	3	24	35	4	FABP1	
RAMP3	Receptor (G protein-coupled) activity modifying protein 3	AGCTTGTGGCCTCTATC			1,000	29	0	34		RAMP3	
EMCN	Endomucin	CTACTTTGTACATATAA	TTTTCTTTAA	361	449	13	0	34		EMCN	
RAPGEF3	Rap guanine nucleotide exchange factor (GEF) 3	AGGAGGGGCTGGGACTG			184	6	72	33	3	RAPGEF3	
FAM20B	Major histocompatibility complex, class I, B	TCCCGGCCCGGCCGCGG			82	2	0	33		FAM20B	
NPNT	Nephronectin	GTAAAGGTATAAGCCTT	CATTTTTAAT	383	286	9	0	32		NPNT	Ref:NPNT
UACA	Uveal autoantigen with coiled-coil domains and ankyrin repeats	AGTTCTGTTTCACAAGT			82	3	0	32		UACA	
KLK7	Kallikrein-related peptidase 7	AGCCACCACGGCCAGCC			592	19	96	31	6	KLK7	
LIMS2	LIM and senescent cell antigen-like domains 2	CAGATGGAGGCCTCTGG			776	25	24	31	32	LIMS2	
TYRO3	TYRO3 protein tyrosine kinase	GGGCGGGTCCTAGCTGT			1,143	37	72	31	16	TYRO3	

Transcripts represented by SAGE tags enriched 30 fold or more in glomeruli compared to pooled SAGE libraries from diverse tissues and cells. The gene symbol and gene name are shown for each tag sequence. Where available, the corresponding short Sau3A SAGE tag (Ref 9) is shown. SAGE tag frequencies are shown as Tags per Million (TPM). Sau3A TPM is derived from Ref 9. Enrichment in glomeruli relative to pooled libraries (G:P) and relative to whole kidney (G:K) is shown for each tag. This table with embedded links to SAGE Genie and links to references showing expression and/or function in glomeruli is provided as additional file 3

**Table 2:** Transcripts represented by SAGE tags enriched 10 – 30 fold in glomeruli

Symbol	Gene Name	NlaIII long Tag	Sau3A Tag	G:TPM Sau3A	G:TPM NlaIII	P:TPM NlaIII	K:TPM NlaIII	Ratio G: P	Ratio G: K	Link SAGE Genie	Ref Link
COL4A4	Collagen, type IV, alpha 4	TAACTTTTGCAAGATGC			122	4	0	29		COL4A4	Ref:COL4A4
AQP1	Aquaporin 1	AGCTCCTGATCAGAGGC			102	4	0	27		AQP1	AQP1
NPR1	Natriuretic peptide receptor A/guanylate cyclase A	AGCAGAGACAATTAAAA			163	6	24	27	7	NPR1	NPR1
PTGDS	Prostaglandin D2 synthase 21kDa	ACGGAACAATAGGACTC	CCGGCCAGCC	5,323	2,368	88	24	27	99	PTGDS	
CRHBP	Corticotropin releasing hormone binding protein	AATAAATACATTCAGAA	ATAGTTCTAA	654	286	11		27		CRHBP	
TNNI1	Troponin I type 1 (skeletal, slow)	AGGCACCTGGGGCTTCT	TGCGGGCCAA	158	204	8	24	26	9	TNNI1	
ODZ2	Odz, odd Oz/ten-m homolog 2	ACAGTCACCACGAGGAG			122	5		26		ODZ2	
EPAS1	Endothelial PAS domain protein 1	GAACTTTTCTGTAATGG			82	3	48	24	2	EPAS1	
PODXL	Podocalyxin-like	GAGGACACAGATGACTC	ATATATGTCT	2,323	3,369	141	48	24	70	PODXL	Ref:PODXL
TMEM204	Transmembrane protein 204	CGTGCGAGACACGTGTG			204	9		23		TMEM204	
SMAD6	SMAD family member 6	TCTCCGGACGCCACCAA			163	7		23		SMAD6	Ref:Samd6
CLEC3B	C-type lectin domain family 3, member B	ACCGGCGCCCGCATCGC	GTGTAGCCGG	361	367	16	72	23	5	CLEC3B	
IFITM3	Interferon induced transmembrane protein 3	AACCCCTGCTGCCTGGG			102	4		23		IFITM3	
HOXA7	Homeobox A7	GTATGTTGTCTTGAGTT			82	4		21		HOXA7	
CHI3L1	Chitinase 3-like 1 (cartilage glycoprotein-39)	GTATGGGCCCTGGACCT	CCCAAGCCTG	564	1,021	48		21		CHI3L1	
FRY	Furry homolog (Drosophila)	TGAACTTGTTGCACTGC			408	19		21		FRY	
PEA15	Phosphoprotein enriched in astrocytes 15	TCTGCCCTTTTTTGTGG			125	6	24	21	5	PEA15	
ZNF250	Zinc finger protein 250	TGGAACCACAAGCAGCC			143	7		20		ZNF250	
TGFBR3	Transforming growth factor, beta receptor III	GCAAATCCTGTCGGTCT	CTCCTGTCTA	180	122	6		20		TGFBR3	
IGFBP5	Insulin-like growth factor binding protein 5	GAGTACGTTGACGGGGA	TTTGTCTTTT	3,496	367	18		20		IGFBP5	Ref:IGFBP5
FCN3	Ficolin (collagen/fibrinogen domain containing) 3	GACACCGAGGGGGGCGG	GTCAGCCACC	180	715	36	48	20	15	FCN3	
LRRC32	Leucine rich repeat containing 32 (GARP gene)	TTGCATACCCTGACCCC	TTTGAAAACA	180	20	1		19		LRRC32	
NOSTRIN	Nitric oxide synthase trafficker	CCACACGCAGATTCACT	TTTGAATGGG	158	61	3		19		NOSTRIN	Ref:Nostrin
HTRA1	HtrA serine peptidase 1	TTTCCCTCAAAGACTCT			1,409	74	24	19	59	HTRA1	
ITGA1	Pelota homolog (Drosophila)	TGCCAGGTGCAGTCACA			122	6	24	19	5	ITGA1	
TNNC1	Troponin C type 1 (slow)	TCCTCAACCCCAAATCC			184	11		17		TNNC1	
DOPEY2	Dopey family member 2	AGAATTGCTTGAACCCA			1,347	79	263	17	5	DOPEY2	
FOXC1	Forkhead box C1	AGCCTGTACGCGGCCGG	ATTGTTAAAG	519	245	15	24	17	10	FOXC1	
CEP3	CDC42 effector protein (Rho GTPase binding) 3	ATGCTTCTGCAGAGACT	TTGGGCCCTC	226	163	10		17		CEP3	
BGN	Biglycan	GCCTGTCCCTCCAAGAC	GAGAACGGGA	158	776	48	72	16	11	BGN	Ref:BGN
MAGI2	Membrane associated guanylate kinase	TATTAATAGTCACAGAA			102	7		16		MAGI2	
AIF1L	Allograft inflammatory factor 1-like	GGAGTGTGCGTGGACTG			1,572	102	334	15	5	C9orf58	
KCNH3	Potassium voltage-gated channel, subfamily H, member 3	TGCCCCTGCCTCTACCT			163	11		15		KCNH3	
PARD6G	Par-6 partitioning defective 6 homolog gamma (C. elegans)	TCGTTCAGTGCCCCAGC			82	5		15		PARD6G	
TM4SF18	Transmembrane 4 L six family member 18	CAAGTATACCACCCTTC			82	5		15		TM4SF18	
TENC1	Tensin like C1 domain containing phosphatase (tensin 2)	AATAGGGGAAAAAAGAG	ACATGAATAG	519	531	37		15		TENC1	Ref:TENC1
DPP6	Dipeptidyl-peptidase 6	ACATTTGGTTAAAAAAA			82	6		14		DPP6	
NES	Nestin	TGCTGACTCCCCCCATC			2,001	138	72	14	28	NES	Ref:NES
ADORA1	Adenosine A1 receptor	TGACTAATAAAAAACTG			82	6		14		ADORA1	Ref:ADORA1
VEGFA	Vascular endothelial growth factor A	TTTCCAATCTCTCTCTC	CCCTGGCTCC	248	1,450	109	24	13	61	VEGFA	Ref:VEGFA
ZNF135	Zinc finger protein 135	GGGAAACTCCATCTCTA			102	8		13		ZNF135	
SLC48A1	Solute carrier family 48 (heme transporter), member 1	GTGCATCAGAGCGGGAA			82	6		13		SLC48A1	
FARP1	FERM, RhoGEF (ARHGEF) and pleckstrin domain protein 1	GGGTAGTGTCAGTCGGA			122	10	72	13	2	FARP1	
C1orf115	Chromosome 1 open reading frame 115	TCGAAGATTCACTGGGA			102	8	24	12	4	C1orf115	
DAG1	Dystroglycan 1 (dystrophin-associated glycoprotein 1)	CAGAGACGTGGCTGGCC			1,286	104	48	12	27	DAG1	Ref:DAG1
PPAP2A	Phosphatidic acid phosphatase type 2A	AAACACCAACAACTGGG	CAGATTGGTC	248	286	23		12		PPAP2A	Ref:PPA2A
SEMA3B	Semaphorin 3B	TGCCGCCCGCAGCCTGC			612	50	72	12	9	SEMA3B	
CDC14A	CDC14 cell division cycle 14 homolog A (S. cerevisiae)	TATTTTGTTATGAATAG			143	12	24	12	6	CDC14A	
CIRBP	Cold inducible RNA binding protein	TGCCCGGGGAATGTTCC			82	7		12		CIRBP	
CSRP1	Cysteine and glycine-rich protein 1	CAGGCGGGGTCCTAGGA			82	7		12		CSRP1	
FAM20C	Family with sequence similarity 20, member C	CGCCCGTCGTGAATTCA			367	31	119	12	3	FAM20C	
CLEC16A	C-type lectin domain family 16, member A	CTTCGTGGGTACTGAAC			122	10	24	12	5	CLEC16A	
INF2	Inverted formin, FH2 and WH2 domain containing	TCCAGCCCCTGAAGTTG			184	16	48	12	4	INF2	
BMP7	Bone morphogenetic protein 7	TGGAACCCGGTCTTGTG			204	18		12		BMP7	Ref:BPM7
ZDHHC6	Zinc finger, DHHC-type containing 6, transmembrane protein 4	TGGTACTTCTCTTTTCC	AATGGATGTT	1,128	592	51		12		ZDHHC6	
PPAP2B	Phosphatidic acid phosphatase type 2B	ATGTAGGTGCCACCCAC	AACCACATGC	654	184	16	119	11	2	PPAP2B	Ref:PPA2B
TXNIP	Thioredoxin interacting protein	AGAAACTAGAGGGCAGG			102	9		11		TXNIP	
ITGA3	Integrin, alpha 3	GTACTGTAGCAGGGGAA	CTCCACAGAG	180	817	72	24	11	34	ITGA3	Ref:ITGA3
C19orf63	Chromosome 19 open reading frame 63	AAAGAGTCGGGGCTGGA			82	7	48	11	2	C19orf63	
IGFBP2	Insulin-like growth factor binding protein 2, 36kDa	GCCTGTACAACCTCAAA	CAGGGAGCCC	880	1,797	161	96	11	19	IGFBP2	Ref:IGFBP2
PTGER4	Prostaglandin E receptor 4 (subtype EP4)	TTTTGTTGCTCAGTGTT			306	28		11		PTGER4	
FLRT3	Fibronectin leucine rich transmembrane protein 3	TATTTTTCTAGGCATAA			82	8	24	11	3	FLRT3	
VWA1	Von Willebrand factor A domain containing 1	CCCAGGACACCAGCTGG			531	49	24	11	22	VWA1	
LARGE	Like-glycosyltransferase	AAAGCCCAGTTCTGAAG			82	8	24	11	3	LARGE	
LINGO1	Leucine rich repeat and Ig domain containing 1	AAGATGATATGAGGCCG			122	12		10		LINGO1	
MYO1E	Myosin IE	TATGAATGTACTAAGTA	ATATACTGTA	248	245	26		9		MYO1E	Ref:MYO1E

Transcripts represented by SAGE tags enriched 10 – 30 fold in glomeruli compared to pooled SAGE libraries. The gene symbol and gene name are shown for each unique NlaIII-anchored long SAGE tag sequence. Where available, the corresponding short Sau3A SAGE tag (Ref 9) is shown. SAGE tag frequencies are shown as Tags per Million (TPM). Sau3A TPM is derived from Ref 9. Enrichment in glomeruli relative to pooled libraries (G:P) and relative to whole kidney (G:K) is shown for each tag. This table with embedded links to SAGE Genie and links to references showing expression and/or function in glomeruli is provided as additional file 4

A previously published Sau3A-anchored SAGE library [9] prepared from microdissected human glomeruli contained 184 SAGE tags that were enriched in glomeruli relative to other micro-dissected nephron segments. These represented 156 well-characterized transcripts. As expected, the corresponding NlaIII SAGE tag for 143 of these was also observed in the current glomerular SAGE library (Tables 1 and 2 and Additional files 3 and 4 and additional file 2). For 47 transcripts represented in both libraries a 10-fold or greater enrichment of the NlaIII tag relative to non-glomerular cells and tissues was observed and is shown in Tables 1 and 2 and Additional files 3 and 4. The NlaIII tag corresponding to the remaining 96 transcripts identified in the Sau3A library was enriched relative to whole kidney, in keeping with the previous report [9], but less than 10 fold relative to non-renal tissues (additional file 2).

Many of the highly expressed and highly enriched transcripts observed in this library are encoded by genes already known to be unique or highly enriched in glomerular podocytes, for instance Podocin (NPHS2), Nephrin (NPHS1), transcription factor 21 (Pod1, FLJ35700), Protein Tyrosine Phosphatase Glepp 1 (PTPRO), Synaptopodin (SYNPO), indicating that this SAGE database appropriately represents glomerular transcripts and that it identifies transcripts enriched in glomeruli. Some of the SAGE tags enriched in glomeruli represent known endothelial cell-predominant genes, for instance Endomucin (EMCN), claudin 5 (CLDN5), NOSTRIN and CD34, consistent with abundant EC in glomeruli.

To independently demonstrate the utility of this database in defining enrichment of transcripts in glomeruli, RT-PCR comparing the level of expression of 19 transcripts enriched in the glomerular SAGE library with that in lung, spleen and liver was performed. Lung, liver and spleen were not represented in the pooled SAGE libraries used here. For each, glomeruli microdissected from the kidneys of three distinct donors were used. The source mRNA used for RT-PCR was distinct from that used for generation of the SAGE library. Transcripts were chosen to represent a spectrum of glomerular enrichment, and some well-known podocyte-predominant transcripts (TCF21, VEGFA) were included as internal controls. Overall, the degree of glomerular transcript enrichment observed by RT-PCR compared to lung, liver and spleen was similar to that observed by SAGE, though there was variation between lung, spleen and liver (Table 3). The wide range of expression observed in the three non-glomerular tissues was expected, as the pooled SAGE-based comparison does not take into account tissue-to-tissue variation in gene expression.

**Table 3:** Ratio of mRNA abundance in glomeruli compared to lung, spleen and liver.

			SAGE			RT-PCR		
Symbol	Name	LongTag	G: TPM	P: TPM	Ratio G: P	G: Lu	G: Sp	G: Li
SOST	Sclerosteosis	ACATATGAAAGCCTGCA	82	0.00	Infinity	130 ± 48	13007 ± 4827	7969 ± 2957
TCF21	Transcription factor 21	CGAGTGCTGAGCAAGGC	817	1.59	514	49 ± 16	27 ± 9	384 ± 123
FGF1	Fibroblast growth factor 1 (acidic)	TAAAGGCCTTTAATAAG	102	0.49	210	490 ± 70	166 ± 23	2565 ± 367
SPOCK2	sparc/osteonectin, cwcv and kazal-like domains	TGTGGAGTGTACTTGTT	245	1.44	170	45 ± 13	39 ± 12	258 ± 75
PTHR1	Parathyroid hormone receptor 1	TGACCAGGCGCTGGGGG	1143	8	141	196 ± 55	51 ± 14	30 ± 8
EFNB1	Ephrin-B1	AGGGAAGAGGAAAGTGC	102	1.50	68	20 ± 4	17 ± 3	30 ± 6
CDKN1C	Cyclin-dependent kinase inhibitor 1C (p57, Kip2)	TAGCAGCAACCGGCGGC	1021	46	22	51 ± 17	45 ± 15	198 ± 66
IGFBP5	Insulin-like growth factor binding protein 5	GAGTACGTTGACGGGGA	367	18	20	10 ± 4	22 ± 9	43 ± 17
CLDN5	Claudin 5	GACCGCGGCTTCCGCCG	715	38	19	5 ± 1	27 ± 7	24 ± 6
FAM65A	Family with sequence similarity 65, member A	GGTTCCTGGTGCCCCTT	755	43	17	18 ± 5	11 ± 3	51 ± 15
FOXC1	Forkhead box C1	AGCCTGTACGCGGCCGG	245	15	17	25 ± 6	302 ± 74	452 ± 110
C9orf58	Chromosome 9 open reading frame 58	GGAGTGTGCGTGGACTG	1572	102	15	36 ± 3	2 ± 0	171 ± 16
NES	Nestin	TGCTGACTCCCCCCATC	2001	138	14	22 ± 6	23 ± 6	339 ± 84
VEGFA	Vascular endothelial growth factor A	TTTCCAATCTCTCTCTC	1450	109	13	16 ± 4	53 ± 12	22 ± 5
ZDHHC6	Zinc finger, DHHC-type containing 6	TGGTACTTCTCTTTTCC	592	51	12	Infinity	60 ± 18	Infinity
MYO1E	Myosin IE	TATGAATGTACTAAGTA	245	26	9	11 ± 3	21 ± 6	13 ± 3
MYL9	Myosin, light chain 9, regulatory	GGAGTGTGCTCAGGAGT	3287	454	7	4 ± 1	15 ± 4	45 ± 11
ITM2B	Integral membrane protein 2B	TCACCTTAGGTAGTAGG	3328	508	7	4 ± 1	4 ± 1	5 ± 1
MYO1D	Myosin ID	ATTGTAGACAATGAGGG	327	82	4	3 ± 0	7 ± 1	7 ± 1

The SAGE tag frequency in glomeruli (G: TPM) and SAGE library pool (P: TPM) as well as the relative SAGE tag enrichment in glomeruli (Ratio G: P) is shown. The transcript abundance relative to lung (G: Lu), spleen (G: Sp) and liver (G: Li) was determined by RT-PCR using distinct sets of microdissected glomeruli from kidneys of three different donors. G: glomeruli, Lu: lung, Sp: spleen, Li: liver. Mean ± SEM.

Finally, it is of note that 117 transcript tags observed 2 or more times and enriched > 500 fold in this glomerular library remain unidentified or poorly characterized (additional file 1). At least some of these will likely prove to be currently unknown glomerulus-predominant transcripts.

### In Silico Interrogation of the Glomerular SAGE Database

The current database can be retrieved directly or interrogated in silico. It may be used to determine whether any specific gene is highly expressed in glomeruli, and to define transcripts that are highly enriched relative to other tissues for which SAGE libraries are available.

To assess whether a specific transcript is expressed in glomeruli, the SAGE tags uniquely identifying the transcript can be found at http://cgap.nci.nih.gov/SAGE/ using the "SAGE Anatomic Viewer" [21]. The "Digital Northern" tool is then used to evaluate the level of expression in the SAGE libraries of the collection, which includes the current library. The collection can also be interrogated using specific NlaIII SAGE tags of cDNA sequences for which a gene symbol may not yet have been assigned. The tag can be retrieved from any cDNA sequence by identifying the 17-nt sequence immediately 3' of the last NlaIII site (CATG) prior to the poly(A^+^) tail. Its frequency in the glomerular database is an indicator of the level of expression in human glomeruli. The 95% confidence interval for observing any tag with a true count of 4 is ± 3.96. Hence, any transcript producing a tag frequency of 4 per 48,905 (81.8 TPM) or greater has a 95% probability of being represented in this library. Failure to find the SAGE tag representing any specific transcript in this library indicates that its expression level is lower than the limit of detection, or that the transcript does not contain an NlaIII restriction site from which a SAGE tag could be generated.

The "LSAGE_Kidney_Glomeruli_Normal_B_bjballer1" database can also be compared directly to a single, or sets of other SAGE databases in the SAGE Genie collection using the "SAGE Digital Gene Expression Displayer (DGED)" tool at http://cgap.nci.nih.gov/SAGE/. This type of analysis will return data similar to those in additional file 1, though comparison can also be restricted to specific libraries rather than the pool of libraries evaluated here.

Finally, this SAGE library with matching transcript identification, glomerulus to pool ratio and glomerulus to kidney ratio is supplied as additional file 1, where the order is based on tag abundance. This data set contains only 18,152 SAGE tags, as any tag found only once and not in any other library was removed. The table can be retrieved without restriction and, if desired, sorted based on the degree of tag enrichment.

## Discussion

This study established a human glomerular SAGE library that can be used for data mining by investigators with an interest in glomerular cell biology and pathophysiology. The library was appropriately enriched in SAGE tags representing transcripts known to be restricted to glomerular podocytes, including nephrin [24], podocin [25], synaptopodin [26], podocalyxin [27], transcription factor 21 [28], the protein tyrosine phosphatase receptor type O GLEPP1 [29], the cyclin dependent kinase inhibitor C1 [30] and nestin [31]. It is therefore likely that other transcripts whose SAGE tags are much more highly represented in this library compared to SAGE libraries from other tissues and cells are also expressed predominantly in glomeruli.

A SAGE library that used Sau3A as the anchoring restriction enzyme was previously produced from human glomerular mRNA [9]. It identified 155 highly expressed transcripts in glomeruli that were enriched in glomeruli when compared to microdissected non-glomerular nephron segments. Since the previously published glomerular SAGE library is based on the Sau3A anchoring restriction site, it does not allow in silico comparison of tag frequencies with the much greater collection of NlaIII-based SAGE libraries. All except 12 transcripts reported to be enriched in glomeruli by Chabardes-Garonne [9] were observed in the current glomerular SAGE library. The corresponding NlaIII tag for a subset of these (47 tags) was enriched > 10 fold when compared to non-renal tissues and cells (Tables 1 and 2 and Additional files 3 &4), providing independent evidence that these represent glomerulus-predominant transcripts.

The current study also identified 86 transcript tags that were enriched more than 10 fold in glomeruli, but which were not represented in the previous Sau3A anchored library (Tables 1 and 2 and Additional files 3 &4). Failure to find a Sau3A SAGE tag for known glomerulus-restricted genes like nephrin, or an NlaIII SAGE tag for endoglin and VCAM1, suggests either that the tag frequency was too low to be detected or that the required restriction site was absent from the transcript. The current study also shows that that several transcripts more highly expressed in glomeruli compared to other nephron segments [9] are not restricted to glomeruli when compared to non-renal tissues or cells (additional file 2). This is not surprising since some transcripts that are not shared between nephron epithelium and glomerular capillary tuft nevertheless may be highly expressed in other tissues.

Several transcripts not previously shown to have a specific function in glomeruli were highly expressed and enriched in glomeruli when compared to non-glomerular tissues. Among these, the tag for the chloride intracellular channel 5 (CLIC5) is very abundant in the glomerular transcript pool, and its frequency in glomeruli was more than 800 fold greater than in other tissues. The transcript "DKFZp564B076" whose SAGE tag was previously shown to be enriched in microdissected glomeruli [9] and later in cultured glomerular EC in this laboratory [22] is identical to the 3' end of CLIC5. CLIC5 is an ezrin-binding protein involved in maintaining actin-based microvilli in the placenta and actin-based stereocilia in the inner ear [32]. Its role in glomerular cell function is as yet undefined. The transcript for the basal cell adhesion molecule (BCAM) is also very abundant in glomeruli and enriched approximately 58 fold. BCAM is a glycoprotein that functions as a receptor for alpha5 laminin. BCAM immunoreactivity is observed in both, glomerular podocytes and glomerular EC, and mice deficient in BCAM have significant structural abnormalities of glomeruli [33]. Glomerular expression of the parathyroid hormone receptor 1 (PTHR1) was not expected. PTHR1 is very abundant in renal proximal tubule cells and could therefore represent proximal tubule contamination. However, since the PTHR1 SAGE tag was less abundant in renal cortex than in glomeruli (Table 1 and Additional file 3), its enrichment in this library cannot be due to proximal tubule contamination. Indeed, mesangial cells express PTHR1 [34]. More work is required to define the function of PTHR1 in mesangial cells. In this regard, it is of great interest that Sclerostin, an inhibitor of bone matrix formation whose expression is regulated by PTH, is also expressed at much higher levels in glomeruli than in most non-renal tissues and cells (Table 1 and Additional file 3) or in other nephron segments [9]. While we have no comparison with a bone SAGE library where sclerostin is likely expressed at high levels, the finding nonetheless suggests that it could be involved in regulating extracellular matrix depositon in glomeruli. Nephronectin, a ligand for integrin alpha8beta1 is known to be essential for renal development, and is expressed in renal epithelium. Enrichment of the nephronectin SAGE tag in the glomerular library relative to kidney cortex is in keeping with the observation by Brandenberger et al [35], who observed very strong nephronectin immunoreactivity in differentiating glomeruli. The secreted glycoprotein testican 2 SPARC (SPOCK2) belongs to in the osteonectin/SPARC family [36] is also highly expressed and enriched in glomeruli. Members of this family of proteins regulate cell-cell and cell-matrix interactions, and SPOCK2 is induced after glomerular injury [37]. The other protein in this family is connective tissue growth factor (CTGF). The SAGE tag for CTGF was observed at a high frequency in glomeruli (additional file 1) but it was not highly enriched relative to other tissues. Nonetheless, both SPOCK2 and CTGF likely play a critical role in regulating glomerular remodeling. In 2006 Lakhe-Reddy and coworkers [38] described the localization of beta 8 integrin to glomerular mesangial cells and observed that its expression may suppress mesangial cell dedifferentiation via Rac1 activation. The SAGE tag for integrin beta 8 was highly expressed and enriched in this glomerular library.

While several semaphorins are expressed in renal glomeruli, so far a role for semaphorin 3G, whose SAGE tag is abundant and enriched in this database, has not been described. Still, semphorin 3G, which has repulsive function via neuropilin 2 binding in the CSN neuronal guidance, is also highly expressed in kidney [39], begging the question whether it serves an important function is in glomeruli. Based on this study many other transcripts are highly enriched in glomeruli. It is hoped that other investigators will use this database as a tool to further define the transcriptome of glomerular cells in health and disease.

We did not observe the NlaIII SAGE tag for EHD3, a transcript previously shown to be unique for glomerular endothelial cells [12], in this library. A SAGE tag for EHD3 also is not observed in the previously published Sau3A-anchored library [9]. Failure to observe this tag does not detract from the previous observations but only suggests that the EHD3 transcript abundance was too low to generate a SAGE tag in the two glomerular SAGE libraries.

Finally, not all tags observed in this SAGE library have as yet been matched to a specific gene. For some of these unidentified SAGE tags, matching sequences within the human genome are observed, but whether they represent specific transcripts is currently not known.

## Conclusion

We have constructed a new human glomerular SAGE library, based on the NlaIII anchoring restriction site. The database can be searched to determine whether specific transcripts are highly expressed and/or enriched in glomeruli and it can be used a resource to further study transcripts that appear to be glomerulus-enriched but whose function in glomeruli has not been investigated so far.

## Availability and requirements

The SAGE database (GEO Accession #GSM199994) described here is available for download from http://www.ncbi.nlm.nih.gov/geo/. It can also be downloaded from, or interrogated in silico at http://cgap.nci.nih.gov/SAGE/ without restriction. The annotated database containing Tag sequences, glomerular frequencies, gene identification, as well as frequency ratios to pooled and kidney libraries is available as additional file 1.

## Abbreviations

SAGE: Serial Analysis of Gene Expression; TPM: Tags per million; RT-PCR: Reverse Transcriptase Polymerase Chain Reaction; WT-1: Wilms Tumor 1; EC: Endothelial Cell(s); EST: Expressed Sequence Tag; cDNA: Complementary DNA; bp: base pair.

## Authors' contributions

WF, JN and BJB developed the conceptual design of the study. Contributions by WF and JN were equivalent. WF isolated human glomeruli and prepared RNA from glomeruli for construction of the SAGE library, and performed work shown in Figure 1. AG microdissected human glomeruli for RT-PCR analysis. JN performed RT-PCR on microdissected human glomeruli. SK performed immunofluorescence analysis. BJB performed the bioinformatics work and wrote the manuscript. All authors read and approved the final manuscript.

## Pre-publication history

The pre-publication history for this paper can be accessed here:

http://www.biomedcentral.com/1471-2369/10/13/prepub

## Supplementary Material

Additional file 1 **Annotated NlaIII-anchored human glomerular long SAGE library**. Additional file 1 contains all (18,152) NlaIII-anchored long SAGE tags observed in the current glomerular library and at least twice in this + currently reported long SAGE libraries. The SAGE tags are sorted by frequency (a measure of expression of the corresponding transcript). Genes matched to each tag, chromosomal localization, absolute SAGE tag count in the glomerular library, frequency (TPM) in the glomerular and the pooled libraries and the ratio of glomerular: pooled frequencies are shown. Absolute counts and TPM for the corresponding short NlaIII-based SAGE tag in whole kidney and the ratio of SAGE tag TPM in glomeruli: kidney are shown. The dataset can be downloaded without restriction. Click here for file

Additional file 2 **SAGE tags observed NlaIII and Sau3A glomerular libraries but enriched less than 10 fold**. This dataset represents all Sau3A tags reported by Chabardes-Garonne (9) to be enriched in glomeruli when compared to microdissected nephron segments that were also observed in the current NlaIII anchored library, but for which the corresponding NlaIII tags were enriched less than 10 fold when compared to pooled non-renal tissues and cells. Click here for file

Additional file 3 **Transcripts identfied for SAGE tags enriched 30 fold or more in Glomeruli compared to pooled SAGE libraries from diverse tissues and cells.** The gene symbol and gene name are shown for each tag sequence. Where available, the corresponding short Sau3A SAGE tag (Ref 9) is shown.  SAGE tag frequencies are shown as Tags per Million (TPM).  Sau3A TPM is derived from Ref 9.  Enrichment in glomeruli relative to pooled libraries (G:P) and relative to whole kidney (G:K) is shown for each tag. Click here for file

Additional file 4 **Transcripts name for SAGE tags enriched 10 - 25 fold in Glomeruli compared to pooled SAGE libraries.**  The gene symbol and gene name are shown for each unique NlaIII-anchored long SAGE tag sequence. Where available, the corresponding short Sau3A SAGE tag (Ref 9) is shown.  SAGE tag frequencies are shown as Tags per Million (TPM).  Sau3A TPM is derived from Ref 9.  Enrichment in glomeruli relative to pooled libraries (G:P) and relative to whole kidney (G:K) is shown for each. tag. Click here for file
